# Association between Statins Administration and Influenza Susceptibility: A Systematic Review and Meta-Analysis of Longitudinal Studies

**DOI:** 10.3390/v16020278

**Published:** 2024-02-10

**Authors:** Fan Wu, Congcong Wang, Shunran Li, Ying Ye, Mingting Cui, Yajie Liu, Shiqiang Jiang, Jun Qian, Jianhui Yuan, Yuelong Shu, Caijun Sun

**Affiliations:** 1 School of Public Health (Shenzhen), Sun Yat-sen University, Shenzhen 518107, China; wufan63@mail2.sysu.edu.cn (F.W.); wangcc5@mail2.sysu.edu.cn (C.W.); lishr53@mail2.sysu.edu.cn (S.L.);; 2 Nanshan District Center for Disease Control and Prevention, Shenzhen 518000, China; 3 NHC Key Laboratory of System Biology of Pathogens, Institute of Pathogen Biology, Chinese Academy of Medical Sciences, Peking Union Medical College, Beijing 100730, China; 4 Key Laboratory of Tropical Disease Control (Sun Yat-sen University), Ministry of Education, Guangzhou 514400, China

**Keywords:** statins, influenza, susceptibility, infection risk

## Abstract

Previous studies reported that the association between statins use and influenza infection was contradictory. A systematic review and meta-analysis of longitudinal studies were performed to determine the association between statins use and influenza susceptibility. The literature search was conducted in PubMed, Embase, and Web of Science, from each database’s inception to 21 May 2023. The fixed effect model and random effects model were used for data synthesis. In our study, a total of 1,472,239 statins users and 1,486,881 statins non-users from five articles were included. The pooled risk ratio (RR) of all included participants was 1.05 (95% CI: 1.03–1.07), and there were still significant differences after adjusting for vaccination status. Of note, RR values in statins users were 1.06 (95% CI: 1.03–1.08) in people aged ≥60 years old and 1.05 (95% CI: 1.03–1.07) in participant groups with a higher proportion of females. Administration of statins might be associated with an increased risk of influenza infection, especially among females and elderly people. For those people using statins, we should pay more attention to surveillance of their health conditions and take measures to prevent influenza infection.

## 1. Introduction

Statins, a class of HMG-CoA (hydroxymethylglutaryl CoA) reductase inhibitors, are widely used for the primary and secondary prevention of cardiovascular and cerebrovascular disease [1], which are ranked first in various causes of death worldwide. It is well known that statins are extensively used as first line agents to reduce the level of low density lipoprotein cholesterol (LDL-C) [2]. Apart from their role in regulating blood lipids, they have been shown to play roles in anti-thrombotic and anti-inflammatory functions. In recent years, statins have been among the most prescribed medicines, especially in the United States, China, India, and many other countries. In 2018, an estimated 145.8 million people were using statins all over the world [3]. Considering the increasingly large population using statins, it is of great significance to investigate how statins administration affects a host’s immune system, and especially their anti-infective function.

A host’s immune system serves as a powerful protective barrier against pathogens infection [4]. The first line of immune response is innate immunity, which is primarily formed by macrophages, antigen-presenting cells (APCs), innate lymphoid cells (ILCs), and a variety of non-immune cells. Innate immunity is initiated via the recognition of conserved pathogen-associated molecular patterns (PAMPs) by cellular pattern recognition receptors (PRRs) in host cells [5,6]. Invading nucleic acids, such as viral RNA or DNA, are recognized by different PRRs, which trigger signaling pathways and ultimately induce the expression of type I interferons (IFN-Is), proinflammatory cytokines, and other antiviral effector genes [7,8,9]. However, some proinflammatory cytokines caused by uncontrolled viral replication may result in an excessive inflammatory response, and eventually lead to tissue damage [10].

There are two strategies to deal with viral infection through host innate immunity: elimination of the virus or reduction of the negative impact of infection [11]. Statins can decrease NF-κB activation in macrophages, exhibit anti-inflammatory properties by reducing both the prenylation of signaling molecules with downregulation of gene expression and the expression of adhesion molecules, as well as reducing levels of cytokines and chemokines [12,13]. In theory, during later stages of viral invasion, statins can reduce tissue damage by suppressing cytokine storms; this has attracted many clinical trials currently underway [14]. Meanwhile, in the early stages of viral invasion, type I interferon production and NF-κB activation are necessary for the host to eliminate the virus [15]. Some downstream effectors inhibit viral replication and promote activation of the adaptive immune response, leading to antiviral immune responses [16]. Several studies have found that statins can affect the expression of type I interferon, although the exact target has not been determined [17,18,19]. Therefore, statins may act as a regulator of innate immunity affecting early viral infection.

As one of the most important pathogens of respiratory infections, the influenza virus poses a severe challenge to global public health, and accounts for 290,000–650,000 annual influenza-associated deaths worldwide [20]. To date, studies have been investigating the impact of statins use on the effectiveness of influenza vaccines and the morbidity or mortality of influenza infection, but the association between statins use and influenza susceptibility has been contradictory in different studies. For example, some studies reported that mortality in patients hospitalized with influenza was significantly lower in statins users than it was in statins non-users [21]. However, other studies showed that statins use was associated with an increased risk of laboratory-confirmed influenza [22] and reduced influenza vaccine efficacy against medically attended acute respiratory illness (MAARI) [23]. In addition, some studies found that there was no statistically significant difference in the risk of hospitalization for pneumonia or influenza between statins users and non-users [24,25]. It is urgent to perform a comprehensive analysis of conflicting results from the above studies to clarify which results might be due to the period of statins use, the state of the users, and the definition of treatment outcomes.

Incidence rate is an important indicator to evaluate the new infection situation of a virus in the population. In the present study, we conducted a systematic review and meta-analysis, based on all published longitudinal studies, to determine the association between statin use and the incidence of influenza infection, and explored the potential influencing factors of this association. This study aimed to provide insights into guiding the rational use of statins in different populations with various health conditions.

## 2. Materials and Methods

This systematic review and meta-analysis was conducted following the recommendations of PRISMA guidelines [26,27]. The study protocol was registered in PROSPERO, the international prospective register of systematic reviews (CRD42023414747).

### 2.1. Literature Search and Selection Criteria

In this study, we searched PubMed, Embase, and Web of Science for articles published in English from each database’s inception to 21 May 2023, using the following terms: (‘statin’ OR ‘atorvastatin’ OR ‘rosuvastatin’ OR ‘simvastatin’ OR ‘pitavastatin’ OR ‘lovastatin’ OR ‘fluvastatin’ OR ‘pravastatin’ OR ‘Hydroxymethylglutaryl-CoA reductase inhibitors’ OR ‘HMG-CoA reductase inhibitors) AND (‘influenza’ OR ‘flu’). Longitudinal studies were included if they contained the number of confirmed influenza infections in statins users and non-users. Individuals infected with influenza were defined as those who had been diagnosed with influenza using nucleic acid detection or rapid influenza diagnostic tests. Exclusion criteria consisted of (1) preprint articles; (2) reviews, systematic reviews, and meta-analyses; (3) research at the cellular or animal level; and (4) articles including participants who did not use statins before they were infected with influenza.

### 2.2. Data Extraction and Quality Assessment

Two researchers (F.W. and C.W.) independently reviewed all potentially relevant studies for eligibility, and then extracted the basic information. The following data were extracted from each study: the author’s name, publication date, study design, study period, country/region, participants’ characteristics, diagnostic method, and sample sizes of groups of exposed individuals (statins users) and control individuals (statins non-users). The number of people infected with influenza in statins users and statins non-users groups in each study were extracted. The quality of studies included in our analysis was assessed using the Newcastle–Ottawa Quality Assessment Scale (NOS), which contained 3 domains: selection, comparability, and outcomes [28]. The full NOS score is 9, and an article with a score of 7 or higher is rated as good quality, a score of 5 to 6 as moderate quality, and 4 or below as poor quality [29]. Details of the quality assessment can be found in Supplementary Tables S1 and S2. Any disagreements between the two researchers (F.W. and C.W.) were resolved by consulting a third senior researcher (C.S.).

### 2.3. Statistical Analysis

We performed a meta-analysis to estimate the pooled risk ratio (RR) and its 95% confidence interval (CI) for the incidence of influenza infection diagnosed by laboratory detection in statins users compared to statins non-users. According to the recommended synthetic calculation methods [30], if the included articles were less than five, we used the fixed effect model (also called the common effect model) to calculate pooled values regardless of the heterogeneity. As for the calculation for more than five articles, we selected synthetic models based on the heterogeneity assessment, which was calculated using I^2^ statistics. An I^2^ statistic greater than 50% was classified as substantial heterogeneity, and then the random effects model was used for the synthetic calculation. In contrast, the fixed effect model was applied when the I^2^ statistic was ≤50%. The effect of publication bias for included articles was quantitatively assessed by Egger’s and Begg’s tests. To verify the robustness of our results, we conducted a sensitivity analysis by excluding each article through each update calculation. The influences of potential confounding factors were assessed by subgroup analyses of participants’ ages, sex, and health conditions.

All data synthesis and analysis were performed using R, version 4.1.1 (R Foundation for Statistical Computing, based in Vienna, Austria). All *p* values were two-tailed, and a *p* value less than 0.05 was considered to be significant.

## 3. Results

### 3.1. Characteristics of Included Studies

**Figure 1:**
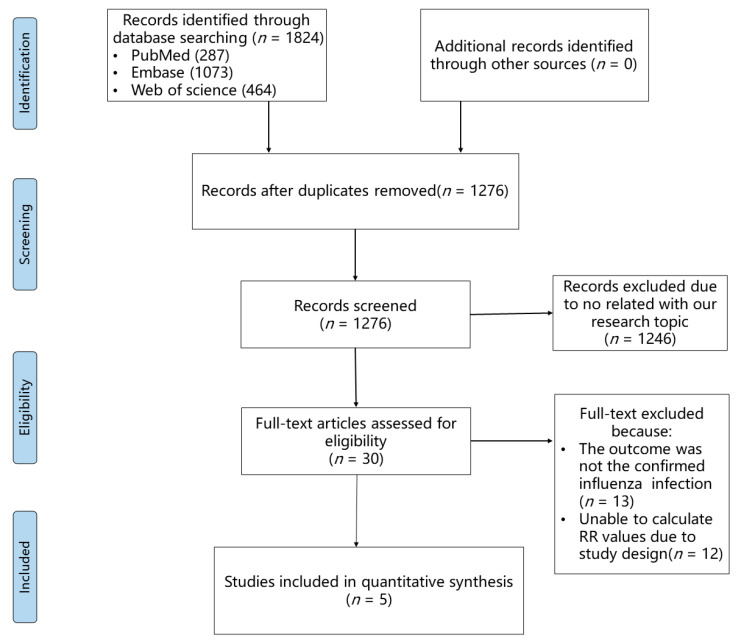
PRISMA flow diagram of the study selection process.

A total of 1824 records were yielded through our search strategy. After preliminary selection and removal of duplicates, full texts of 30 articles were thoroughly reviewed. After a thorough review, 13 articles were excluded because their outcomes were not consistent regarding confirmed influenza virus infection, and 12 articles were eliminated due to an uncomputable RR value. Finally, five articles [22,31,32,33,34] were included in our meta-analysis. The flowchart of literature screening is shown in Figure 1. Among these included studies, 1,472,239 statins users and 1,486,881 statins non-users from three countries (Canada, Australia, and the USA) were observed for the outcome of nucleic acid detection or rapid diagnostic tests for influenza (Table 1). Two articles (Chung [22] and Izurieta [31]) only selected older people (aged >65/66 years old) as participants, and the other three articles included young people as participants. The earliest study we included was conducted during the 2004–2005 influenza season, and the most recent one was conducted during the 2018–2019 influenza season. Three articles were cohort studies, which allowed us to directly calculate RR values. Although two articles were termed as case-control/test-negative studies, we could still indirectly calculate RR values because they were all prospective designs. All included studies had good or satisfactory quality as assessed by the Newcastle–Ottawa Quality Assessment Scale. Details of the quality assessment can be found in Supplementary Tables S1 and S2.

### 3.2. Association between Statins Administration and Influenza Infection

**Figure 2:**
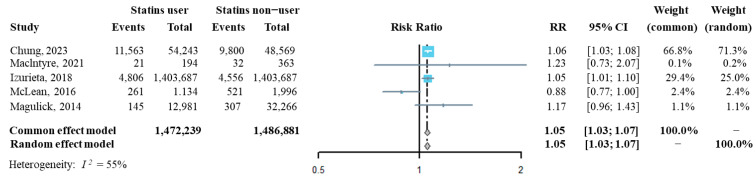
Forest plot of risk ratio of overall statins administration on the risk of influenza infection [22,31,32,33,34].

The influenza incidence from each of the above five studies was extracted, and then we synthesized the risk ratio (RR) of influenza infection in statins users compared to that of statins non-users. As shown in Figure 2, the pooled RR of influenza infection by analyzing with both the fixed effect model and the random effect model was significantly higher in statins users than it was in statins non-users (RR = 1.05, 95% CI: 1.03–1.07), suggesting that statins administration might increase the risk of confirmed influenza infection. The sensitivity analysis indicated that the overall pooled RR was robust (Supplementary Figure S1). Additinally, Egger’s and Begg’s tests showed that publication bias had a small influence on the pooled result (t = −0.25, *p* = 0.819 by Egger’s test; z = 0.24, *p* = 0.807 by Begg’s test, Supplementary Figure S2).

To further explore potential influencing factors associated with statins administration and influenza susceptibility, we performed subgroup analysis according to participants’ influenza vaccination status. After matching for participants’ vaccination status, statins administration remained associated with influenza susceptibility (Supplementary Figure S3).

**Figure 3:**
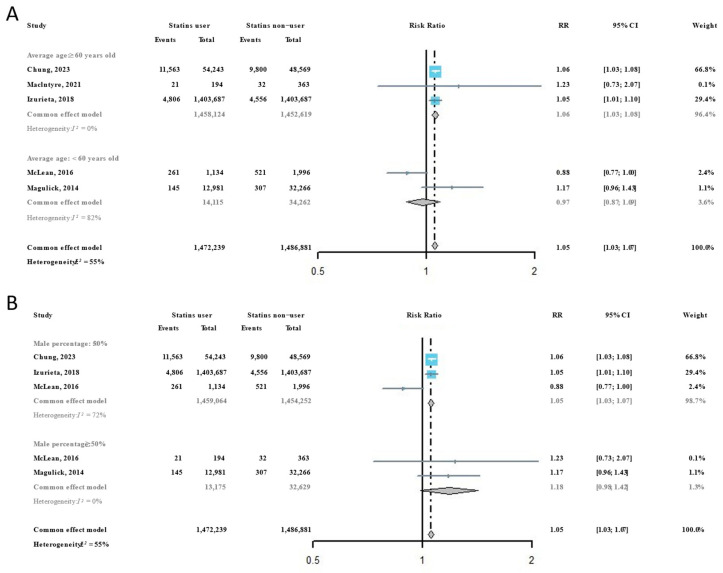
Subgroup analysis of the risk ratios of statins administration on the risk of influenza infection [22,31,32,33,34]: (**A**) by average participant age in included studies; and (**B**) by participant sex composition in included studies.

Considering that age and sex are important influencing factors for virus susceptibility, all included studies were subsequently classified according to the average age and female percentage of participants. Among participants with an average age ≥ 60 years, we found that the risk of influenza infection in statins users was significantly higher than it was in statins non-users (RR = 1.06, 95% CI: 1.03–1.08), but this phenomenon was not observed among participants with an average age < 60 years (RR = 0.97, 95% CI: 0.87–1.09) (Figure 3A). For participant groups with a higher percentage of females, the risk of influenza infection in statins users was significantly higher than it was in statins non-users (RR = 1.05, 95% CI: 1.03–1.07), but the RR in participant groups with a higher percentage of males showed no significance differences between statins users and statins non-users (Figure 3B).

### 3.3. Systematic Review of Health Conditions Confounding the Risk of Statins Administration on the Risk of Influenza Infection

In addition to sex and age, participants’ health conditions might also have been an important factor influencing the risk of virus infection, and might have led to participants’ differing drug use. Considering this potential confounding effect, it was necessary to perform a subgroup meta-analysis according to health conditions. However, included studies in this meta-analysis had major differences in their definitions of high-risk conditions that caused a high heterogeneity, and thus they were not suitable for quantitative synthesis. Alternatively, we conducted a systematic review for a qualitative understanding of the effect of health conditions on our results (Table 2). In sum, all included studies considered the effect of health conditions, and then matched or adjusted them during analysis. Moreover, two studies analyzed the effect of health conditions on influenza infection, but their results were inconsistent (Chung [22] and MacIntyre [34]).

## 4. Discussion

The objective of our study was to explore the effect of statins administration on influenza susceptibility, and the main conclusion of this study supports that statins might act as inhibitors of innate immunity and contribute to increasing an individual’s risk of influenza susceptibility. Although the RR value we identified was only 1.05 (95% CI: 1.03–1.07), which suggested that people who use statins have a 5% increased risk of influenza infection compared to those who do not use statins, this slight risk cannot be ignored, because hundreds of millions of people worldwide use statins. In the subgroup analysis, we found that age and sex significantly influenced the risk of influenza infection caused by the administration of statins. The RR values were 1.06 (95% CI: 1.03–1.08) for elderly people (average age ≥ 60 years) and 1.05 (95% CI: 1.03–1.07) for participant groups with a higher proportion of females (male percentage < 50%). However, for participant groups with a high percentage of young people or males, the administration of statins might not have affected the risk of influenza susceptibility. Therefore, our study further demonstrated the discrepancy in influenza susceptibility in different populations by age and sex, which is consistent with previously studies [35,36]. Interestingly, our result is also consistent with another meta-analysis, which indicated that statins administration might increase the risk of herpes zoster virus infection (OR = 1.18, 95% CI 1.11–1.25) [37].

In addition to being first-line agents used to prevent cardiovascular and cerebrovascular diseases, statins have been also found to effectively decrease the hyperinduction of proinflammatory cytokine storms produced by viral infections [38]. As a result, statins have been applied to treat some viral infections, such as COVID-19, but the effectiveness of this treatment in real-world studies was inconsistent [39,40], suggesting that lipid metabolism has different effects on viral infection [41,42,43,44,45] and that statins may not be limited to reducing the hyperinduction of inflammatory responses. Mechanically, in addition to their potential anti-inflammatory effects, previous studies have demonstrated that statins could play multiple roles in the regulation of virus susceptibility. Statins can reduce cholesterol availability, disrupt lipid raft composition, alter membrane receptor assembly, and thus limit virus fusion and entry into host cells [46]. One study also demonstrated that statins reduced the replication of influenza H1N1 by blocking RhoA and LC3 lipidation localization and inducing actin filaments condensation [47]. However, statins treatment showed no prophylactic or beneficial effect in influenza virus-infected mice [48,49]. Some studies even found that statins may attenuate a host’s antiviral function. For example, simvastatin and pitavastatin decreased poly(I:C)-induced IFN-β expression by restraining the phosphorylation of interferon regulatory factor 3 (IRF3) and signal transducers and activators of transcription 1 (STAT1) [50]. Another study showed that simvastatin and atorvastatin reduced the expression levels of IFN-α/β receptors and clathrin-mediated endocytosis, and thus abrogated the host’s response to IFN-α [51]. Thus, these findings suggest that statins may intricately affect interferon signaling pathways, which is a critical factor for the regulation of a host’s antiviral function. In the future, the exact mechanism should be further clarified.

Notably, a previous study assessed the effect of statins treatment on mortality due to influenza virus infection, but not on influenza susceptibility; it found increased survival in patients who were undergoing statins treatment [52]. However, our conclusion supported that statins administration might be associated with an increased risk of influenza infection. Possible explanations for this discrepancy might be as follows: (1) To further validate the causal inference of our conclusion, we included only longitudinal studies to analyze the risk ratio (RR) instead of the odds ratio (OR) used in the former studies. (2) More importantly, we strictly defined the study outcome as laboratory-confirmed influenza virus infection; thus, the interference of other diseases with similar symptoms could be excluded, while the former studies all focused on mortality caused by influenza-like symptoms.

Although these were the strengths of our study, several limitations should be acknowledged. First, although we found a slight association between statin use and influenza susceptibility, the number of included studies was small, which may limit the ability to generalize our conclusions. Second, some of the original studies did not provide the detailed stratification and duration of statins administration, which impeded us from performing a further subgroup analysis to determine other confounders. Third, the number of studies included in our subgroup analysis was relatively low, causing us to use only the fixed effects model for the synthetic calculation. Finally, the included studies were all observational studies, and there was a lack of randomized clinical trials to provide us with further evidence. Therefore, there is an urgent need for further study regarding the impact of statins administration on viral infections.

## 5. Conclusions

In conclusion, statins administration is slightly associated with an increased risk of influenza infection, especially among females and elderly people. Age and sex, rather than the status of influenza vaccination, might change this association. Although we found that the administration of statins may increase the risk of influenza susceptibility, it should be emphasized that we do not encourage people who were prescribed statins to stop taking these drugs just for influenza prevention. Alternatively, we strongly urge people using statins to pay more attention to surveilling their health conditions and take measures to prevent influenza infection while taking statins.

## Figures and Tables

**Table 1:** Characteristics of included studies.

Study	Study Period	Study Region	Study Design	Age	Diagnostic Method	Clinical Characteristics of Patients	Sample Size		Quality
							Statin Users	Non-Users	
Chung, 2023 [22]	2010–2011 to 2018–2019 influenza seasons	Ontario, Canada	Prospective case-control study	≥66 years (Mean age ≥ 60)	Nucleic acid detection, antigen detection, and viral culture	Not applicable	54,243	48,569	Good
MacIntyre, 2021 [34]	2008–2010 winter seasons	Sydney, Australia	Prospective test-negative design	≥40 years (Mean age = 66.3)	Polymerase chain reaction	Influenza with acute myocardial infarction	194	363	Satisfactory
Izurieta, 2018 [31]	2010–2011 through 2014–2015	USA	Retrospective cohort study	>65 years (Mean age ≥ 60)	Rapid influenza diagnostic test	Influenza-related office visits or influenza hospitalizations	1,403,687	1,403,687	Good
McLean, 2016 [32]	2004–2005 to 2014–2015 influenza seasons	Wisconsin, USA	Cohort study	≥45 years (Mean age < 60)	Real-time reverse transcription polymerase chain reaction	Acute respiratory illness	1134	1996	Good
Magulick, 2014 [33]	2010–2011 to 2018–2019 influenza seasons	San Antonio, USA	Retrospective cohort study	35 to 80 years (Mean age = 48.7)	Rapid influenza diagnostic test	Had an outpatient visit and received at least one prescription medication	12,981	32,266	Satisfactory

**Table 2:** Systematic review of included studies’ participants’ health conditions.

Study	Definition of High-Risk Conditions	Whether the Definition Was Matched While Designing the Study or Adjusted during Analysis	Whether the Effect of Health Conditions on Outcomes Were Analyzed	Whether Health Conditions Had a Significant Effect on Outcomes
Chung, 2023 [22]	Anemia, asthma, cancer, ischemic heart disease, arrhythmia, congestive heart failure, chronic kidney disease, COPD, diabetes, dementia/frailty, immunocompromised, history of TIA or stroke	Yes (adjusted during analysis)	Yes	Yes
MacIntyre, 2021 [34]	Diabetes, elevated serum cholesterol, hypertension	Yes (adjusted during analysis)	Yes	No
Izurieta, 2018 [31]	Asthma, bronchitis, liver disease, hypercholesterolemia, hypertension, chronic/acute kidney failure	Yes (matched while designing the study)	No	-
McLean, 2016 [32]	Cardiovascular, diabetes, pulmonary, other high-risk conditions	Yes (adjusted during analysis)	No	-
Magulick, 2014 [33]	A higher mean of Charlson Comorbidity Score	Yes (adjusted during analysis)	No	-

## Data Availability

Datasets for the current study are available from the corresponding author upon reasonable request.

## Supplementary Materials

The following supporting information can be downloaded at: https://www.mdpi.com/article/10.3390/v16020278/s1, Supplementary Table S1. The quality assessment by the Newcastle–Ottawa Quality Assessment Scale (NOS) for cohort studies; Supplementary Table S2. The quality assessment by the Newcastle–Ottawa Quality Assessment Scale (NOS) for case-control studies; Supplementary Figure S1. Sensitivity analysis of risk ratio of overall statins administration on the risk of influenza infection; Supplementary Figure S2. Publication bias tests; Supplementary Figure S3. Subgroup analysis of risk ratio of statins administration matching for participants’ vaccination status.
