# Supplementary material for: Association between Statins Administration and Influenza Susceptibility: A Systematic Review and Meta-Analysis of Longitudinal Studies
Source: Viruses. 2024 Feb 10;16(2):278. doi: 10.3390/v16020278 (PMC10893112; doi:10.3390/v16020278)
Supplement: Supplementary file 1 [file viruses-16-00278-s001.zip › viruses-2838664-supplementary.pdf]

Table S1. The quality assessment by Newcastle-Ottawa Quality Assessment Scale (NOS) for cohort studies.

COHORT STUDIES	Selection				Comparability	Outcome			Score/Quality/
**Studies**	**Q1**	**Q2**	**Q3**	**Q4**	Q5	Q6	Q7	Q8	
Izurieta, 2018	*	*	*	*	**	*	*	*	9(good)
McLean, 2016	*	-	*	*	**	*	*	-	7(good)
Magulick, 2014	*	-	*	*	*	*	*	-	6(satisfactory)

Note: Q1: Representativeness of the exposed cohort; Q2: Selection of the non-exposed cohort; Q3: Ascertainment of exposure; Q4: Demonstration that outcome of interest was not present at start of the study; Q5: Comparability of cohorts on the basis of the design or analysis; Q6: Assessment of outcome; Q7: Was follow-up long enough for outcomes to occur; Q8: Adequacy of follow up of cohorts.

Table S2. The quality assessment by Newcastle-Ottawa Quality Assessment Scale (NOS) for case-control studies.

CASE-CONTROL STUDIES	Selection				Comparability	Outcome			Score/Quality/
**Studies**	**Q1**	**Q2**	**Q3**	**Q4**	Q5	Q6	Q7	Q8	
Chung, 2023	*	*	-	*	**	*	*	-	7(good)
MacIntyre, 2021	-	*	-	*	*	*	*	-	5(satisfactory)

Note: Q1: Is the case definition adequate? Q2: Representativeness of the cases.; Q3: Selection of Controls; Q4: Definition of Controls; Q5: Comparability of cases and controls on the basis of the design or analysis; Q6: Ascertainment of exposure; Q7: Same method of ascertainment for cases and controls; Q8: Non-Response rate.

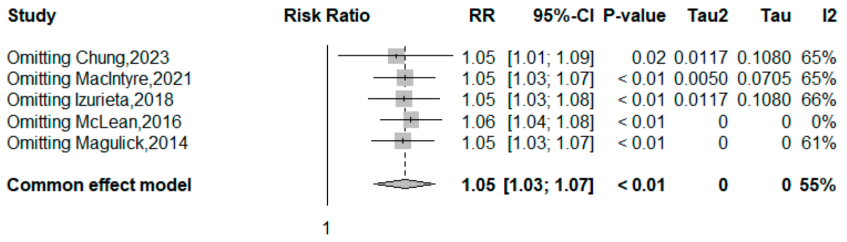

Figure S1. Sensitivity analysis of risk ratio of overall statins administration on the risk of influenza infection.

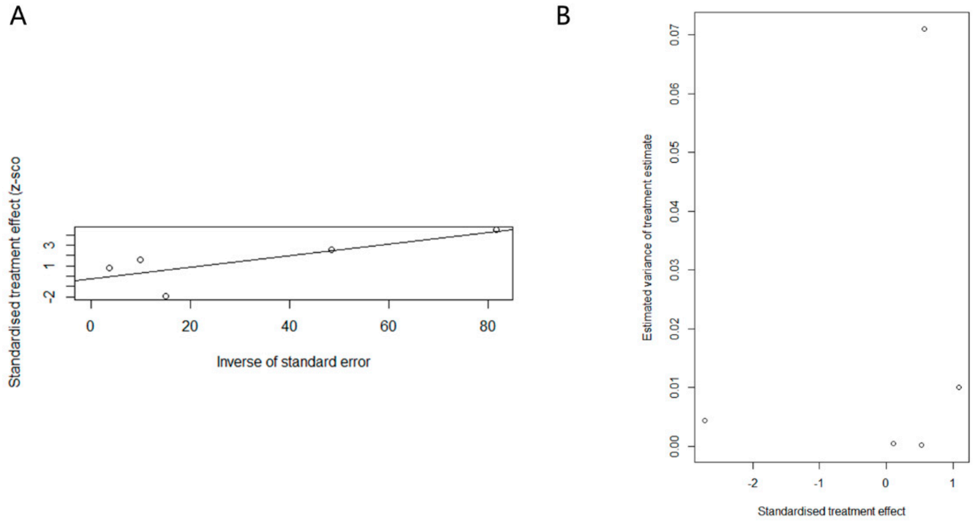

Figure S2. Publication bias tests. (A) Egger's test, t =-0.25，df = 3，p-value = 0.819; (B) Begg's test, z = 0.24, p-value = 0.807.

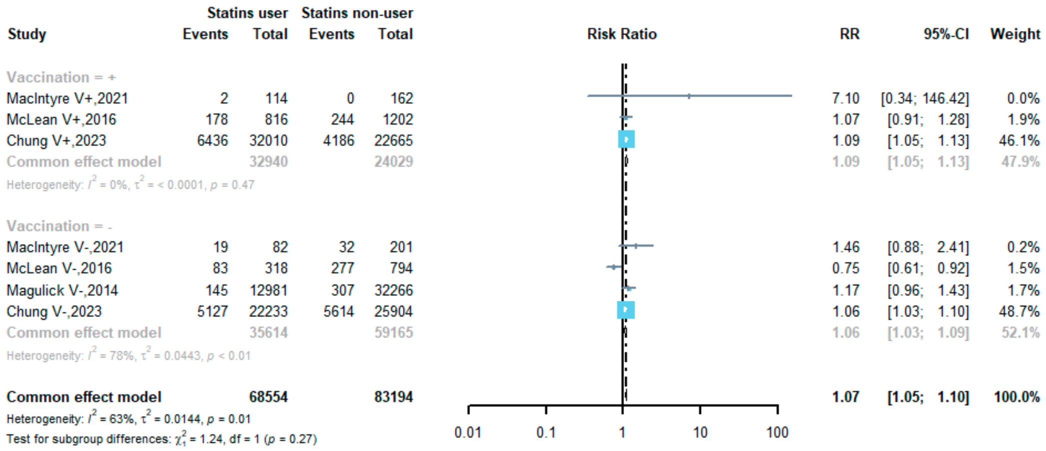

Figure S3. Subgroup analysis of risk ratio of statins administration matching for participants' vaccination status.
